# Fully automating LI-RADS on MRI with deep learning-guided lesion segmentation, feature characterization, and score inference

**DOI:** 10.3389/fonc.2023.1153241

**Published:** 2023-05-18

**Authors:** Ke Wang, Yuehua Liu, Hongxin Chen, Wenjin Yu, Jiayin Zhou, Xiaoying Wang

**Affiliations:** ^1^ First Hospital, Peking University, Beijing, China; ^2^ Department of Precision Diagnosis & Image Guided Therapy, Philips Research, Shanghai, China

**Keywords:** LI-RADS, clinical study, lesion segmentation, feature characterization, problem formulation, deep learning

## Abstract

**Introduction:**

Leveraging deep learning in the radiology community has great potential and practical significance. To explore the potential of fitting deep learning methods into the current Liver Imaging Reporting and Data System (LI-RADS) system, this paper provides a complete and fully automatic deep learning solution for the LI-RADS system and investigates its model performance in liver lesion segmentation and classification.

**Methods:**

To achieve this, a deep learning study design process is formulated, including clinical problem formulation, corresponding deep learning task identification, data acquisition, data preprocessing, and algorithm validation. On top of segmentation, a UNet++-based segmentation approach with supervised learning was performed by using 33,078 raw images obtained from 111 patients, which are collected from 2010 to 2017. The key innovation is that the proposed framework introduces one more step called feature characterization before LI-RADS score classification in comparison to prior work. In this step, a feature characterization network with multi-task learning and joint training strategy was proposed, followed by an inference module to generate the final LI-RADS score.

**Results:**

Both liver segmentation and feature characterization models were evaluated, and comprehensive statistical analysis was conducted with detailed discussions. Median DICE of liver lesion segmentation was able to achieve 0.879. Based on different thresholds, recall changes within a range of 0.7 to 0.9, and precision always stays high greater than 0.9. Segmentation model performance was also evaluated on the patient level and lesion level, and the evaluation results of (precision, recall) on the patient level were much better at approximately (1, 0.9). Lesion classification was evaluated to have an overall accuracy of 76%, and most mis-classification cases happen in the neighboring categories, which is reasonable since it is naturally difficult to distinguish LI-RADS 4 from LI-RADS 5.

**Discussion:**

In addition to investigating the performance of the proposed model itself, extensive comparison experiment was also conducted. This study shows that our proposed framework with feature characterization greatly improves the diagnostic performance which also validates the effectiveness of the added feature characterization step. Since this step could output the feature characterization results instead of simply generating a final score, it is able to unbox the black-box for the proposed algorithm thus improves the explainability.

## 1 Introduction

Cancer is a leading cause of death worldwide, accounting for nearly 10 million deaths in 2020 (1). Liver cancer is the third most common cause of cancer death (accounting for 830,000 deaths) in 2020 according to the World Health Organization (2). Hepatocellular carcinoma (HCC) is the most common type of liver cancer, accounting for approximately 90% of all liver cancers (3), with only 18% with 5-year survival rate and average survival rates between 6 and 20 months. To provide standardization of liver imaging for HCC, Liver Imaging Reporting and Data System (LI-RADS) (4) is created and supported by the American College of Radiology (ACR). The imaging normally refers to either multiphase computed tomography (CT) or multiphase magnetic resonance imaging (MRI). LI-RADS is a comprehensive system for standardizing the terminology, technique, interpretation, reporting, and data collection of liver imaging, diagnosis, and staging of HCC in high-risk patients. By providing standardization, LI-RADS aims to reduce imaging interpretation variability, enhance communication with referring clinicians, and facilitate quality research. According to the LI-RADS system, an observed liver lesion in a high-risk patient is assigned a LI-RADS category from LR-1 to LR-5, which indicates the likelihood of being HCC and the extent of the disease spread. More specifically, LR-1 and LR-2 mean definitely benign and probably benign, respectively. LR-3 indicates an intermediate probability of malignancy. LR-4 and LR-5 represent probably HCC and definitely HCC, respectively.

An accurate LI-RADS category system requires an accurate and comprehensive evaluation of imaging features, such as non-rim arterial phase hyperenhancement (APHE), lesion size, the presence of enhancing capsule and non-peripheral washout, and threshold growth. Although the LI-RADS system standardizes the qualitative diagnosis for HCC and improves the reporting workflow, it requires strong reading and interpretation abilities from experts and still might incur large inter-reader and inter-center variations. The increasing complexity of LI-RADS has made its implementation less feasible in a high-volume practice (5) and becomes a major barrier to broad adoption. Triggered by these motivations, researchers tried to seek assistance from artificial intelligence (AI)-based imaging techniques. It is expected to develop computational decision-support tools to improve workflow efficiency by automating the detection, classification, and standardized reporting of diagnostic results. For example, the American College of Radiology has called for novel systems or tools that can seamlessly integrate LI-RADS into radiologists’ normal workflow to make it more feasible for daily clinical care (6).

To automate the LI-RADS system using deep learning techniques, it is common to think of a convolutional neural network (CNN) as an advanced mapping function for classification tasks between inputs (which are MR images) and outputs (which are LI-RADS grades). Instead of doing like a black box, in this paper, we try to simulate the working principles of radiologists and unbox the black box by actualizing the LI-RADS system step by step including lesion detection and segmentation, discriminative feature characterization, and standardized scoring. These steps need to be undertaken integrally and automatically. Unlike general approaches directly classifying the LI-RADS grades based on MRI images, this paper proposes to introduce one more step, which is called feature characterization, in our deep learning framework. To actualize the feature characterization in a deep learning way, this paper delves into the multi-phase reasoning problem for discriminative lesion feature characterization, and it is tailored for the LI-RADS scoring system. Overall, this paper aims to achieve a deep learning-driven complete solution by automating LI-RADS for liver diagnosis to be classified into three types from LR-3 to LR-5. In addition, the feature characterization as an intermediate result is able to help improve the explainability of the proposed approach. To be more specific, the proposed framework consists of three steps as a complete solution, namely, UNet++-enabled liver lesion segmentation, discriminative lesion feature characterization, and inference module for the final LI-RADS score. Physicians working alongside AI is a type of human-in-the-loop AI, which is envisioned to be the future way of work. The AI-empowered proposed integrated framework helps to improve physicians’ self-confidence when diagnosing so as to improve their final diagnostic performance. To be more concrete, the contributions can be summarized as follows.

**Figure 1:**
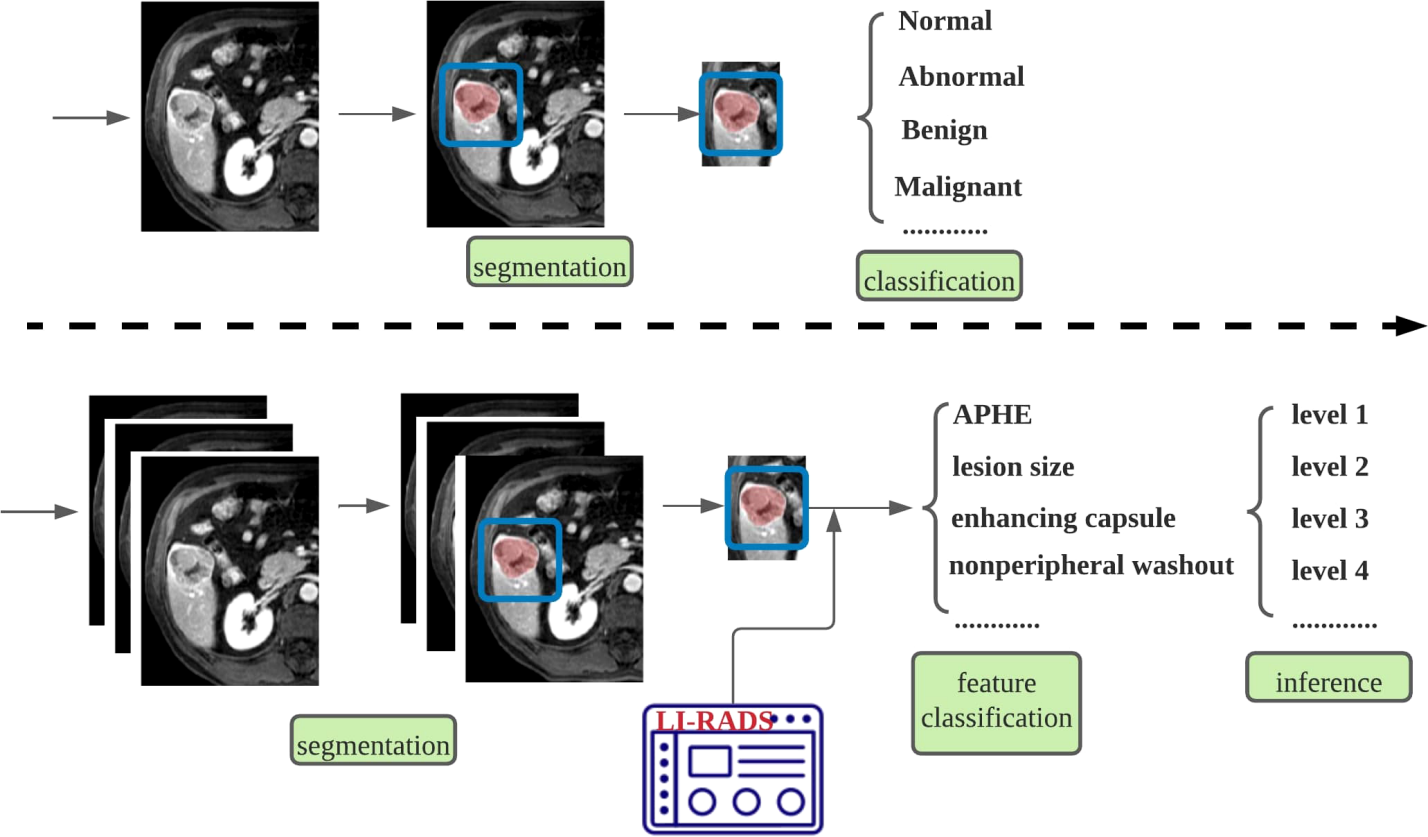
Comparisons of the generally adopted idea of classifying lesion types (top row) and the proposed framework specifically tailored for LI-RADS (bottom row). LI-RADS, Liver Imaging Reporting and Data System.

- This paper not just provides a deep learning-based LI-RADS classification framework but also embeds expert domain knowledge into the deep learning framework and makes an attempt at practical applications of it. In comparison to previous related studies, this paper proposes feature characterization to develop a tailored approach specifically for LI-RADS application instead of simply adopting an existing CNN model to prove the feasibility of deep learning techniques.
- By looking into the working principle of LI-RADS, this paper identifies the types of lesions from a totally new perspective (see **Figure 1**), proposing to characterize the lesion features using a deep learning technique instead of simply treating it as a general classification problem, which directly outputs a LI-RADS grade for each lesion. In this way, the decisions on LI-RADS can be justified through internal analysis of relevant radiologic features, which makes the proposed framework explainable.
- Considering the special characteristics of contrast-enhanced MRI with multiple-phase images being involved, this paper designs a specific network for multi-phase reasoning problems for discriminative lesion feature characterization, and the newly designed architecture can be jointly trained by using multi-task learning with adaptive loss.
- The proposed framework has been tested and investigated in our designed study, and a comparison experiment was also conducted to validate the effectiveness of the added feature characterization step. The comprehensive statistical analysis was performed using R software based on the dataset from Peking University First Hospital (PKU1). Statistical results are discussed in detail to analyze the possible challenges in this study.

## 2 Materials and methods

### 2.1 Deep learning study design

**Figure 2:**
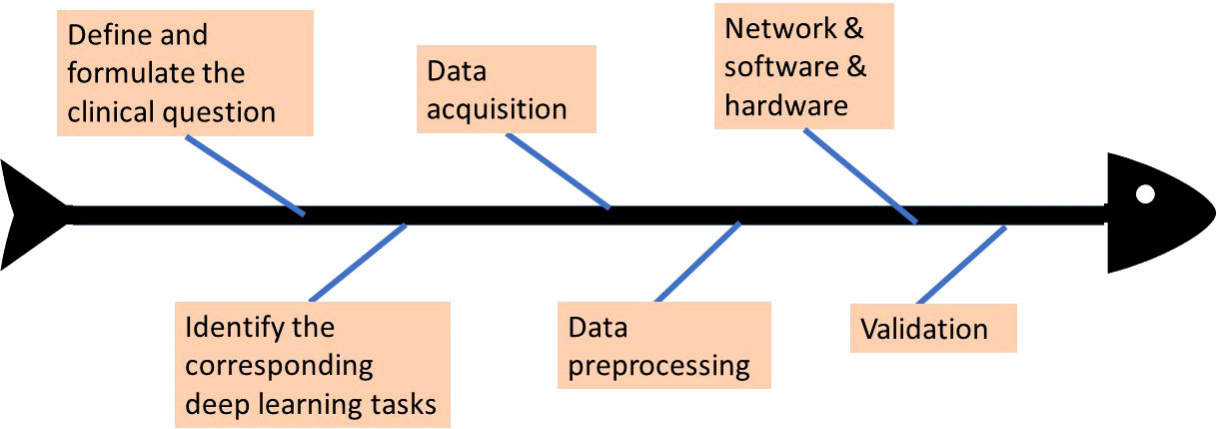
The workflow of deep learning study design.

Deep learning has rapidly advanced in various fields and also gained attention in the radiology community. The paper of Yasaka et al. (7) starts with an introduction to deep learning technology and then presents the stages that are entailed in the deep learning study design process of radiology research. A standard workflow or process of a complete study design is provided. The initial step is the formulation of the clinical problem, which is the LI-RADS scoring system in our case. After determining what the clinical problem is, the corresponding deep learning tasks are assigned. Based on the chosen specific deep learning tasks, data acquisition and data preprocessing can be conducted, including the considerations of the split of training data and testing data, and data annotation. Given the tasks and input data, the network architecture is designed, and appropriate software and hardware platforms need to be selected. With both data and network model well prepared, the designed model is trained using training data, by which the parameters inside the model are updated to be optimal values that can deliver the best training results. Last, the network is required to be validated based on testing data to evaluate how well the designed model performs on this specific task. The above briefly describes the study design process, and the study in this paper would follow the above guidelines and showcase how it is designed properly. **Figure 2** illustrates more intuitively how the whole deep learning study is designed and the workflow. The study in this paper would follow the above guidelines to be designed properly.

### 2.2 Clinical problem formulation and study outline

As mentioned previously in the Introduction section, the purpose of this study is to try to work out a solution and establish a fully automatic framework to help radiologists with LI-RADS scoring. This clinical problem could be formulated to be the prediction of the LI-RADS score based on the patients’ liver image, and this main objective can be accomplished by dividing it into three sub-problems and resolving them separately one by one, namely, liver lesion segmentation, lesion feature characterization, and inference module, to obtain the final LI-RADS score. The clinical problem in this study was identified from the radiologists’ daily working experience and generated from real practical needs. This retrospective clinical study was approved by our institutional review board, and the requirement for written informed consent was waived. The reference number of the institutional review board (IRB) approval for the study is 2017-74. It was conducted based on image data of HCC diagnosis between December 2010 and December 2017 in PKU1. All the acquired data were first preprocessed and then split into several datasets: training sets used for training the segmentation and classification models, validation sets used for fine-tuning (if applicable), and testing sets used for verifying the effectiveness of the trained model.

### 2.3 Data acquisition

**Figure 3:**
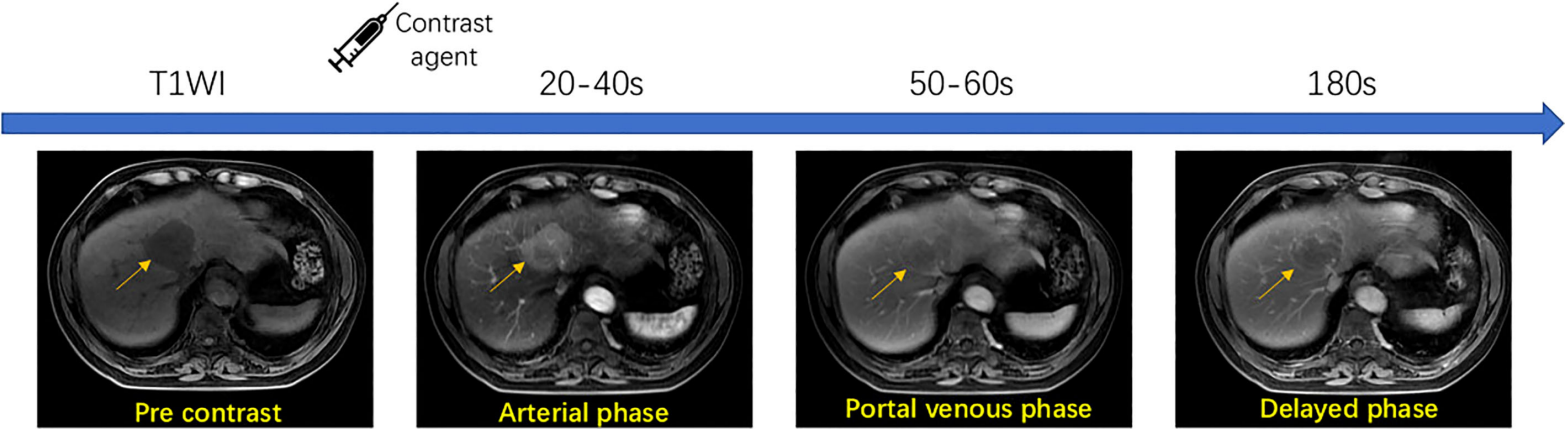
Workflow of multi-phase contrast-enhanced MRI technique.

This study is designed to include a heterogeneous collection of MRI images from different scanners, including SIGNA EXCITE 3.0T HD (GE), Discovery MR 750 3.0T (GE), Achieva 3.0T (Philips), and SIGNA EXCITE 1.5T HD (GE). MRI protocols used are summarized in **Table 1**. Before contrast agent administration, all patients underwent T1-weighted dual-phase sequence, T2-weighted 2D sequence, and diffusion-weighted sequence. After unenhanced imaging, patients received an extracellular contrast medium (Gd-DTPA or Gd-DTPA-BMA) at a rate of 2.5–3 ml/s. A dynamic multiphase T1-weighted (T1W) sequence was acquired 20–40, 50–60, and 180 s after contrast agent injection, during the hepatic arterial, portal venous, and delayed phases, respectively. To demonstrate the multi-phase contrast-enhanced MRI process, **Figure 3** shows a walkthrough example. It can be observed that a typical HCC lesion that the arrow points at is not obvious and non-detectable initially in no contrast phase but gradually appears as a lesion during contrast enhancement, washout, and capsule dynamics in the next three phases. The presence of non-rim APHE, enhancing capsule, non-peripheral washout, and the observation size could be four major features for the determination of LI-RADS grades.

**Table 1:** Imaging sequences and parameters for MRI.

	ST	IG	Matrix	NEX	TR (ms)	TE (ms)	B value
T1-weighted dual-phase FSPGR/FLASH/T1-FFE	≤6	≤1	≥256 × 160	≤1	The shortest	2.25/4.5 (1.5T) 1.15/2.3 (3T)	–
T2-weighted 2D FSE/TSE/SSFSE	≤6	≤1	≥288 × 224	2–4	>1,500	80–106	–
Diffusion-weighted SE-EPI	≤6	≤1	≥128 × 128	4–10	1,850–2,300	The shortest	800–1,200 s/mm^2^
T1-weighted 3D LAVA/THRIVE	≤4.4	0	≥256 × 160	≤1	The shortest	The shortest	–

ST, section thickness; IG, intersection gap; TR, repetition time; TE, echo time; FSPGR, fast spoiled gradient-recalled; FLASH, fast low angle shot; FFE, fast field echo; FSE/TSE, fast or turbo spin echo; SSFSE, single shot fast spin echo; SE-EPI, single shot-echo planar imaging; LAVA, liver imaging with volume acceleration; THRIVE, T1-weighted high-resolution isotropic volume excitation.

**Figure 4:**
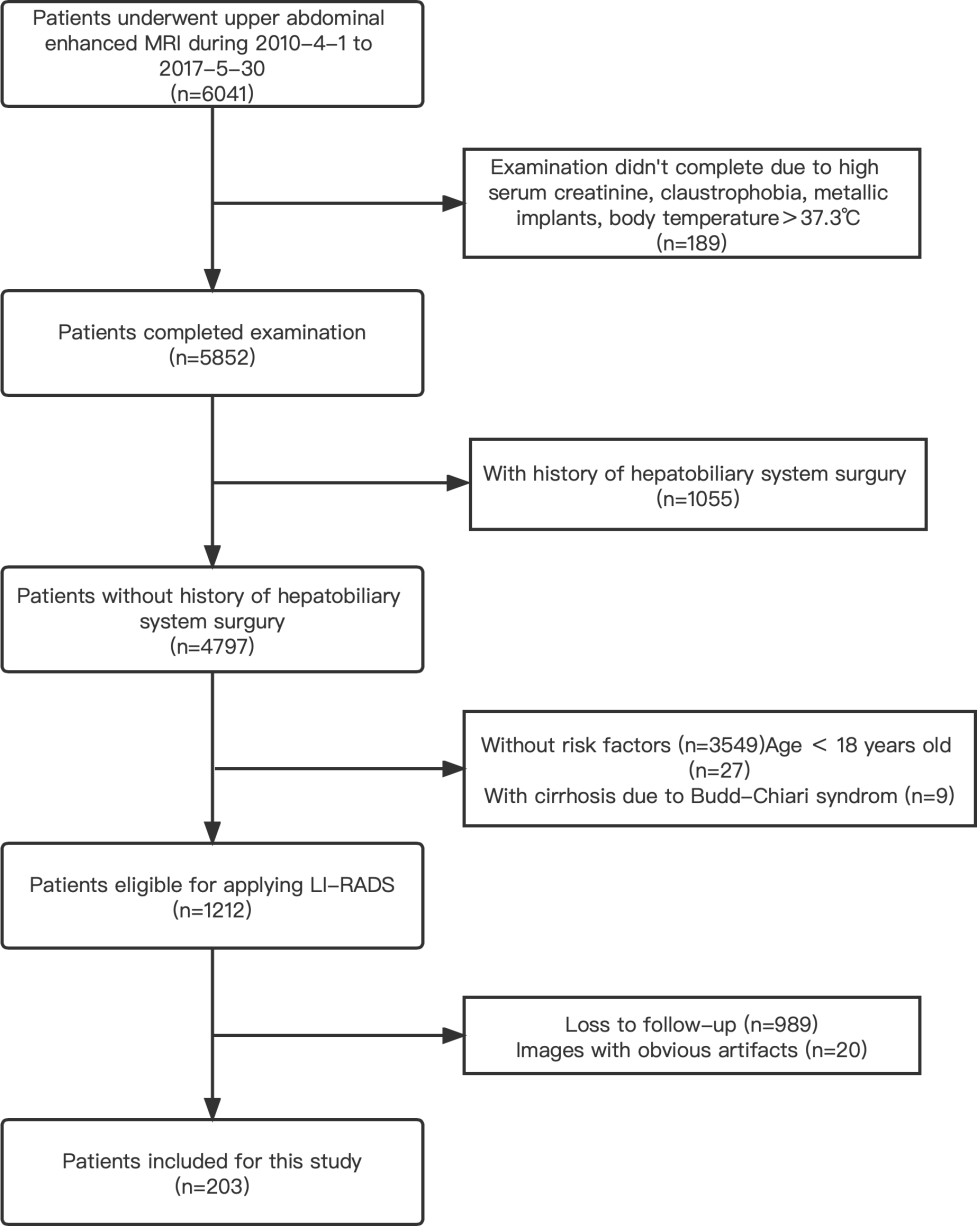
A flowchart of the patient selection process in this study.

A radiologist searched the image archiving and medical history record system for this study performed for the evaluation of focal liver observation (hereafter, we will consistently refer to these as liver lesions unless otherwise specified). A total of 6,041 patients who underwent upper abdomen enhanced MRI examinations and were registered in our institution from April 2010 to May 2017 were included initially. The inclusion and exclusion criteria were detailed in **Figure 4**. The final diagnosis was obtained by pathological tests, transcatheter arterial chemoembolization (TACE) or radiofrequency ablation (RFA), CT/MRI follow-up, or comprehensive clinical diagnosis. Based on these data, the clinical dataset in our study was created and defined as the PKU1 dataset.

All the MRI examinations were evaluated by two experienced radiologists (K.W. and X.W. with 10 and 30 years of imaging experience respectively) according to the LI-RADS 2018 criteria. A liver structure report was generated by the radiologists to record the diagnostic results. Each liver lesion was reviewed, classified, and determined to give a reliable diagnostic result. The lesions were categorized into five classes by referencing radiology reports made by experienced radiologists according to the standardized LI-RADS. The images were annotated with ITK-SNAP software (version 3.6.0; http://www.itksnap.org). The annotation for lesion segmentation and LI-RADS classification were used as the ground truth in statistical analysis.

A total of 203 upper abdomen MRI images obtained in our institution were included in this study (see **Figure 4**). A total of 111 positive patients and 92 negative patients were finally diagnosed (see **Table 2**). The mean age of the participants was 58.53 ± 11.14 years. A total of 69.46% of the patients were male, and 30.54% were female. Of the identified 111 positive patients, 174 liver lesions categorized as LR-3/4/5 were further detected and used as the ground truth. The average diameter of liver lesions was 3.15 ± 2.92 cm, ranging from 0.40 to 16.40 cm. Among all the lesions, 44.8% lesions were smaller than 2 cm.

**Table 2:** Patients diagnosed with HCC (n = 111) and patients with non-HCC (n = 92) and details of how these cases are confirmed.

Diagnostic methods	No. of the positive patients	No. of the negative patients
Liver operation	40	0
Puncture	7	2
Transcatheter arterial chemoembolization	43	0
Radiofrequency ablation (RFA)	4	0
CT/MRI follow-up studies	1	18
Comprehensive clinical diagnosis	14	72
Liver transplant	2	0

### 2.4 Data preprocessing

By using commercial viewing software (Centricity Radiology RA 1000; GE Healthcare), MR images were displayed in Digital Imaging and Communications (DICom) in Medicine format using a window with a size of 512 × 512. For each phase, 300 slices of images were acquired to obtain the 3D shape of the liver, resulting in a 512 × 512 × 300 tensor, which contains 300 images in total with size of 512 × 512.

As the deep learning segmentation model utilizes 2.5D learning technique (will be detailed in Section 2.5.1), a tensor with the size of 512 × 512 × 3 was fed as the input. By doing so, the number of samples that could be used for segmentation would increase to 298 for one patient, resulting in a total of 33,078 (111 image sets × 298) available for segmentation used. Each image was preprocessed by normalization and standardization using the written Python code, and the pixel value of the normalized image $n_{i}$ could be computed by the following:


(1) $$n_{i}=\{\begin{array}{ll} 0, & \text{if}\;o_{i}<80\\ \frac{o_{i}-80}{P_{99}-80}, & \text{if}\;o_{i}\in \left\{80,P_{99}\right\}\\ 1, & \text{if}\;o_{i}>P_{99} \end{array},$$

where $P_{99}$ denotes the 99th percentile. Each pixel is then subtracted by the mean value and divided by the standard deviation for standardization. Unlike CT images, which have a certain range of Hounsfield units (HU) pixel values for each tissue, there would be several pixels with very high signal intensity values in MR images. In addition, from observation, pixels with signal intensity values below 80 always belong to the background; thus, we uniformly replaced signal intensity values below 80 with 0 and values above a threshold with 1 and normalized the values within this range into (0, 1). The threshold used in this study was experimentally set as the 99th percentile of the whole image.

In addition, image augmentation was performed by random rotation, shifting, and flipping both horizontally and vertically to avoid the possible overfitting problem. The generated new images after augmentation were zero-padded to the same size of 512 × 512 as the original images. A ninefold evaluation was performed to verify the effectiveness of the segmentation model used in this study. All the image sets after preprocessing were divided into n folds; thus, the evaluation process was repeated n times. In each iteration, one fold was preserved for testing, and the rest were used to train the segmentation model. n folds take turns as the testing dataset until they all have been evaluated as the testing set. Normally, n could be set as an integer such as 5, 6, 7, 8, 9, and 10, and we randomly chose 9 in this study.

For each phase, the segmentation model can generate 300 image results, and the image with the largest size of the segmented lesion was selected for classification. The segmented region was cropped and then resized into 224 × 224 image to be compatible with VGG16, which has a fixed input size requirement. The resized image was standardized by being subtracted mean value and divided by the standard deviation. After that, the image was also augmented as we did for raw images in the segmentation stage.

### 2.5 Technique: the proposed automatic framework for LI-RADS

**Figure 5:**
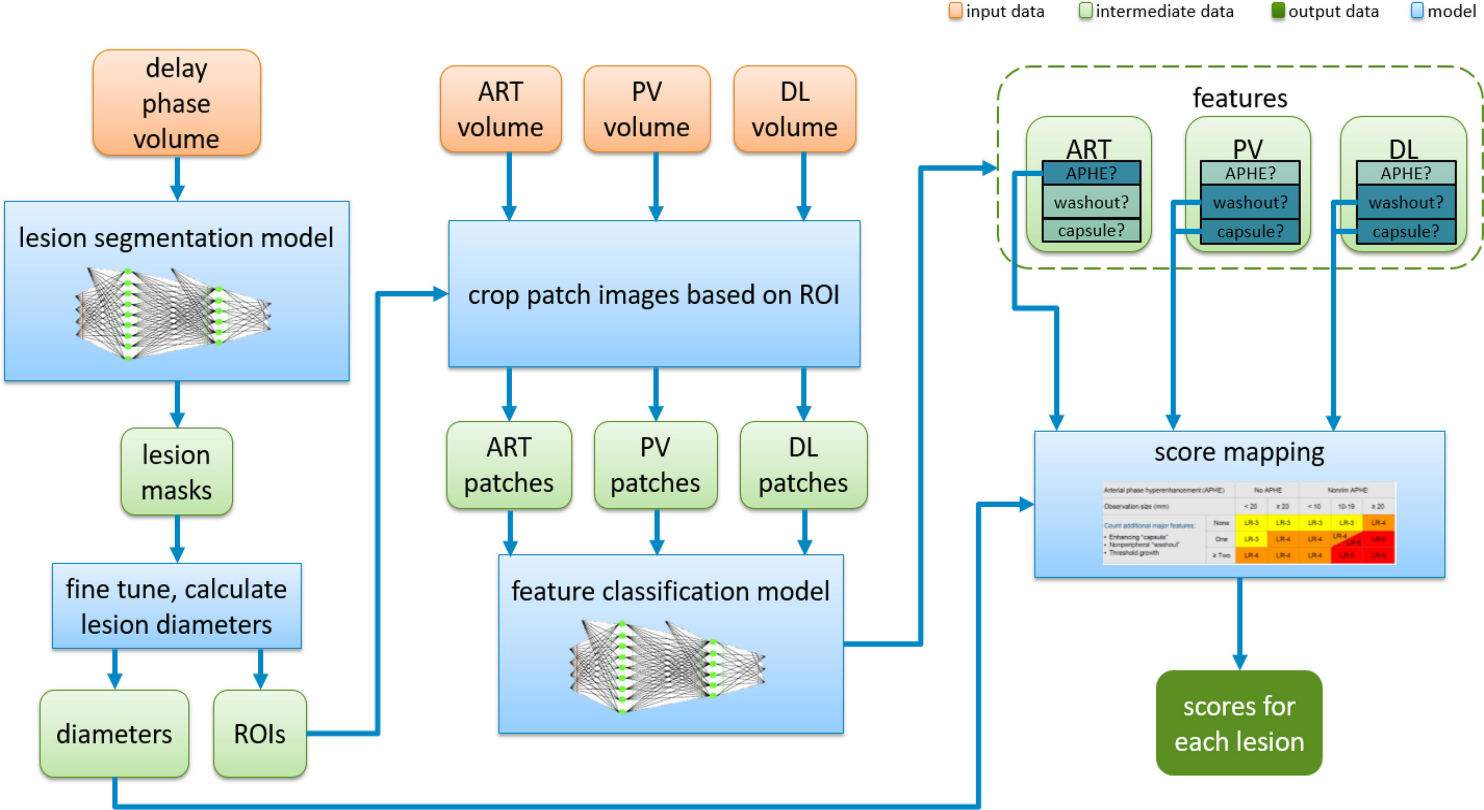
Overview of the fully automatic CNN-guided LI-RADS system. CNN, convolutional neural network; LI-RADS, Liver Imaging Reporting and Data System; ART, arterial phase; PV, portal venous phase; DL, delayed phase; APHE, arterial phase hyperenhancement.

In this section, we will describe in detail the proposed fully automatic framework used for this study for LI-RADS scoring step by step. **Figure 5** shows an overview to provide a big picture of how it works. It can be seen that the proposed system is an integrated end-to-end architecture. Given the inputs of MRI imaging volumes from the three phases, the system directly outputs the final HCC scores for each lesion. Basically, it consists of three parts, which are lesion segmentation, lesion feature characterization, and score mapping. The raw images captured in the delayed phase are first fed into a deep learning segmentation model to automatically extract the region of interest (ROI) that covers the whole lesion instead of cropping manually. Then, the intermediate sub-images from the three phases were treated as the inputs of the characterization model. The characterization model identifies the appearances of APHE, capsule, and washout. On completion of all these four features computation, a specific score could be automatically assigned to each lesion according to the LI-RADS score mapping. In the next subsections, each step in the proposed system will be elaborated on one by one.

#### 2.5.1 Liver lesion segmentation

To precisely extract the liver lesion region of interest, a powerful deep learning model is designed and used by combining UNet++ (8) and convolutional block attention module (CBAM) (9). UNet++ with a ResNet backbone is utilized as the base of the segmentation model, which includes four down-sampling layers for encoding and four up-sampling layers for decoding. On top of that, CBAM is integrated into UNet++ and applied on each residual block for the encoding part. We define a hybrid segmentation loss function consisting of pixel-wise soft dice loss and focal loss (10). When the training loss reaches a predefined threshold, we then add one more Lovasz loss (11) into the loss function for the next training steps doing fine-tuning. Mathematically, the hybrid loss is defined as follows:


(2) $$\begin{array}{l} L\left(y,p\right)\\ =-\frac{1}{n}\sum_{c=1}^{C}\sum_{n=1}^{N}\left(\frac{2y_{n,c}p_{n,c}}{y_{n,c}^{2}+p_{n,c}^{\;2}}+\alpha_{c}{\left(1-p_{n,c}\right)}^{\gamma }y_{n,c}\log\;p_{n,c}\right), \end{array}$$

where $y_{n,c}\in \left\{\pm 1\right\}$ and $p_{n,c}\in \left[0,1\right]$ denote the target labels and predicted probabilities for class c in *n*th pixel in the batch. N indicates the number of pixels within one batch. In our case, c is 2, indicating two classes of lesion and non-lesion. Focal loss is a variant of cross entropy with additions of a weighting factor $\alpha_{c}\in \left[0,1\right]$ for class 1 and 1−α for class −1 and a modulating factor ${\left(1-pn,c\right)}^{\gamma }$ , where γ is the focusing parameter. It is especially useful for class imbalance scenarios. Factor α is introduced to balance the importance of positive and negative examples but not to differentiate between easy and hard examples. This is when modulating factor ${\left(1-p_{n,c}\right)}^{\gamma }$ takes part in down-weight easy examples and only focuses training on hard negatives. When $p_{n,c}$ is small, which means that this pixel is misclassified, the resulting loss should be taken into consideration, and the whole loss function is unaffected. On the contrary, when $p_{n,c}$ is large, it means that this is an easy example, and the resulting loss could be ignored. As $p_{n,c}$ approaches to 1, the factor goes to 0, and the loss for this pixel could be exactly not involved. Parameter γ adjusts the rate at which easy examples are down-weighted. In addition, Lovasz–Softmax loss is defined as follows:


(3) $$L_{add}\left(y,p\right)=\frac{1}{C}\sum_{c=1}^{C}\bar{{\text{Δ}}_{J_{c}}}m\left(c\right))$$

where $\bar{{\text{Δ}}_{J_{c}}}$ is the convex Lovasz extension of intersection-over-union loss.

The integrated deep learning segmentation model is end-to-end trainable with 512×512 preprocessed images as inputs and 512×512 binary images as outputs. Each voxel is predicted as either foreground (lesion region) or background. More specifically, we use 2.5D learning to leverage features in neighboring slices by combing three neighboring slices in a channel as inputs. The designed deep learning model is first trained on the LiTS CT dataset for both liver and lesion segmentation. Then, transfer learning is used to fine-tune the parameters of the pre-trained model based on the PKU1 dataset only for lesion segmentation.

#### 2.5.2 Discriminative lesion feature characterization

**Figure 6:**
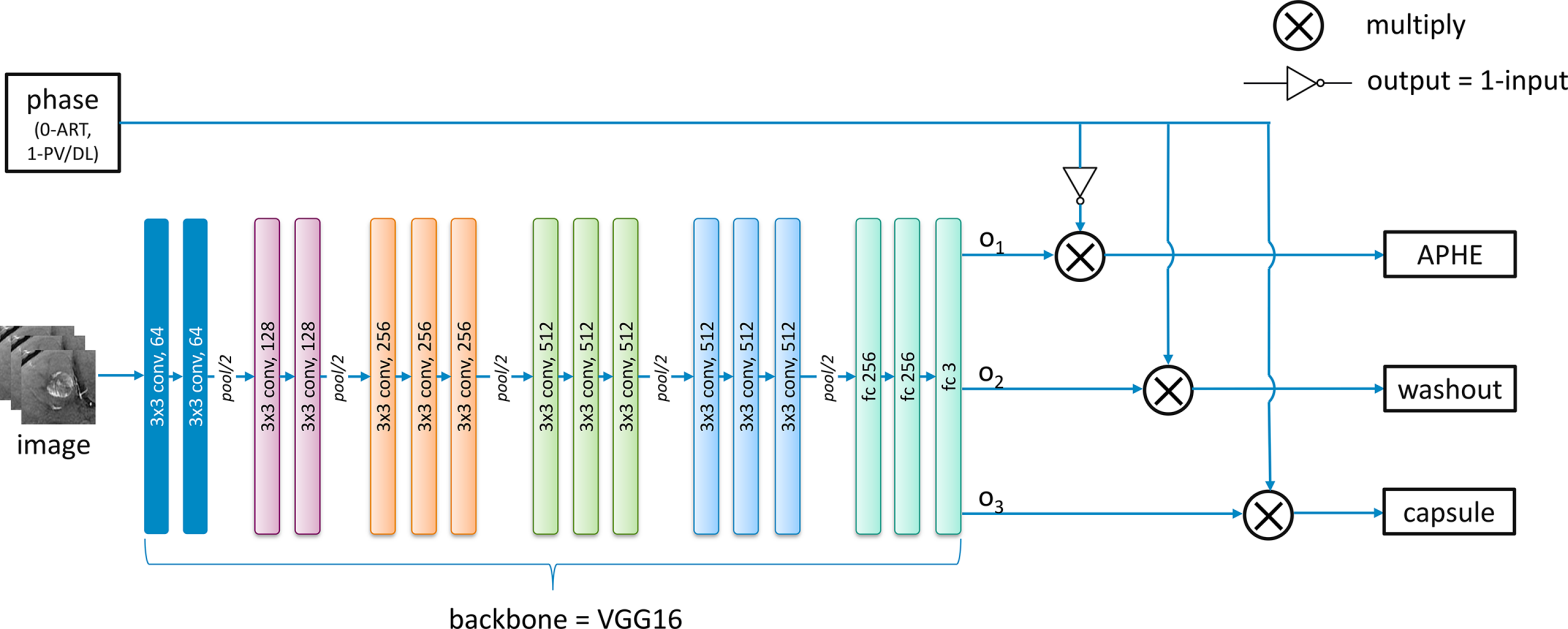
The developed deep learning architecture for the classification of each feature.

**Figure 6** shows the architecture used for the characterization of each feature including APHE, capsule, and washout. Based on the feedback from radiologists, images in the arterial phase can be only used for the determination of APHE, while washout and capsule are inferred from both the portal venous phase and the delayed phase. Although sub-images from different phases are responsible for different features, they are designed to be mixed and combined together to train one shared backbone model in our study as seen in **Figure 5**. Instead of training separated models for different features, only one backbone model is trained due to the lack of sample data to generate a model for the characterization task of each feature, which is responsible for producing a value to represent the possibility of the appearance of each feature. This results in the multi-task learning problem, which is to use one model to resolve multiple tasks. In this study, VGG16 (12) is pre-trained on ImageNet and used as the backbone of our deep learning architecture for lesion feature characterization; then, the model is fine-tuned with inputs of PKU1 dataset based on transfer learning.

For the feature characterization problem in the study investigated in this paper, multi-tasks correspond to the multiple classification problems of different features. However, images from the arterial phase are only responsible for the APHE classification task, and images from both the portal venous and the delayed phases are responsible for both washout and capsule classification tasks. Therefore, we modified the traditional general multi-task learning architecture and incorporated an adaptive loss function into it to achieve the transitions of training loss with regard to images from different phases. The above observations are derived from radiologists’ expert knowledge. During the exploration, we notice that radiologists can explicitly utilize the natural reasoning ability to determine a LI-RADS score by observing different phases. Through the modified multi-task learning with adaptive training, it is able to conduct the joint multi-phase seasoning in our customized model.

For each batch of images from different phases as the input, an additional corresponding input x with 0 or 1 value is also provided and fed into the network. The values 0 and 1 indicate the images are obtained from the arterial phase and the portal venous phase/delayed phase, respectively. The lesion feature characterization model is designed to be embedded with switch operations to achieve the training of the shared parameters. During the training process, when the input image is from the arterial phase (x = 0), the loss function below only considers the first term in which case the network weights are updated only based on feedback on APHE predicted results. On the contrary, the weights are updated only based on the loss on washout and capsule features when the inputs belong to the portal venous phase or delayed phase (x = 1). Mathematically, if the loss function for APHE, washout, and capsule are $L\left(f_{1}\right)$ , $L\left(f_{2}\right)$ and $L\left(f_{3}\right)$ , respectively, the final loss function is defined as follows:


(4) $$L_{all}=\left(1-x\right)L\left(f_{1}\right)+xL\left(f_{2}\right)+xL\left(f_{3}\right),$$

where x represents the input value specified for each phase. Using this adaptive loss function strategy, we do not need to split the dataset according to different phases to train separated models for each feature based on corresponding phases due to the small size of the dataset. Instead, all the images could be used to train a common model, and different features can share the parameters of this model.

#### 2.5.3 Inference module

Once each lesion is detected and segmented and the presence of discriminative features has been identified, the specific LI-RADS score could be inferred according to the following criteria, which is a globally well-accepted gold standard defined by authority. Specifically, the LI-RADS score is derived by the inference rules listed in **Table 3**, where $B_{i}\;\left(i=1,2,3\right)$ is the Boolean indicator that represents the presence of APHE, washout, and capsule. For example, if it is “True” for the presence of APHE, $B_{1}$ is denoted as 1 or 0, which represents “True”, otherwise “False”.

**Table 3:** Inference rules of LI-RADS score based on feature characterization results.

*B*_1_		0 (“False”)		1 (“True”)		
Lesion size		<20	≥20	<10	10–19	≥20
*B*_2_ + *B*_3_	0	LR-3	LR-3	LR-3	LR-3	LR-4
	1	LR-3	LR-4	LR-4	LR-4/5	LR-5
	2	LR-4	LR-4	LR-4	LR-5	LR-5

LI-RADS, Liver Imaging Reporting and Data System.

### 2.6 Implementation details: software and hardware

Section 2 details how the data are acquired and then preprocessed into an analysis-ready format that can be fed into the technical block presented in Section 2.5. Section 2.5 explicitly describes an end-to-end complete process from segmentation, lesion feature characterization, and LI-RADS score inference. The involved three sub-steps can be integrated seamlessly, and the outputs of the previous step would be used as the inputs of the next step. In addition, the related CNN network structures can also be found in this paper or the related references. The whole deep learning framework is implemented in a computer with 128 GB of random access memory, 16 CORE Xeon^®^ Gold 6134 CPU @ 3.20GHz central processing unit (Intel) and a TITAN RTX graphics processing unit (NVIDIA), using Python programming and Keras framework (https://keras.io/) in the Ubuntu 16.04 system.

### 2.7 Deployment and practical use

**Figure 7:**
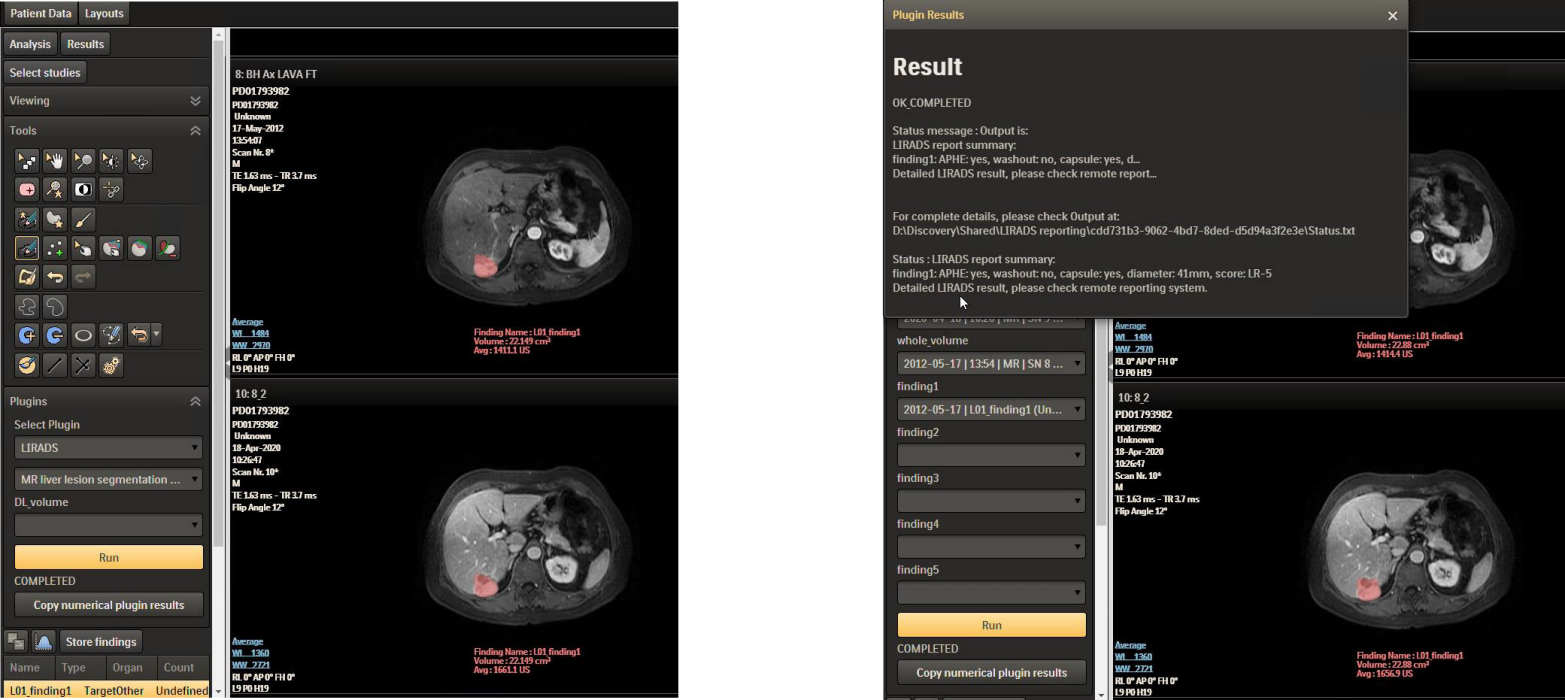
LI-RADS plugin in ISD. LI-RADS, Liver Imaging Reporting and Data System; ISD, IntelliSpace Discovery.

Further, the LI-RADS algorithm could be deployed as a plugin in Philips IntelliSpace Discovery (ISD) platform in PKU1 for further testing and improvement. Philips ISD is an integrated AI research solution that enables the entire process of generating new AI applications, providing data integration, training, and deployment in the research setting. **Figure 7** shows the LI-RADS plugin user interface (UI), where the segmentation result is shown as a mask and the LI-RADS features and score are shown in the result window after clicking the “Run” button. The whole user workflow contains multiple steps rather than a one-click thing. Multi-modules are implemented along the workflow. At the segmentation interface, users can visualize the segmentation results where manual editing is allowed to adjust the interest of the segmented lesion area and then feed them into the next step. When achieving the final LI-RADS scoring interface, the ISD platform also allows users to edit the feature characterization for further score mapping.

## 3 Results

To verify how well the proposed system performs and the effectiveness of the segmentation results, we test it on the PKU1 dataset. Both the segmentation and scoring results have been evaluated and compared with annotated ground truth. We first verify the segmentation quality of liver lesions since the segmentation result could directly affect the measurement of lesion size. In addition, it is the first step of our system, and we cannot have an accurate predicted score of LI-RADS without a good segmentation outcome. Then, we evaluate the lesion feature characterization results by comparing the final predicted score with the radiologist-annotated LI-RADS score.

**Figure 8:**
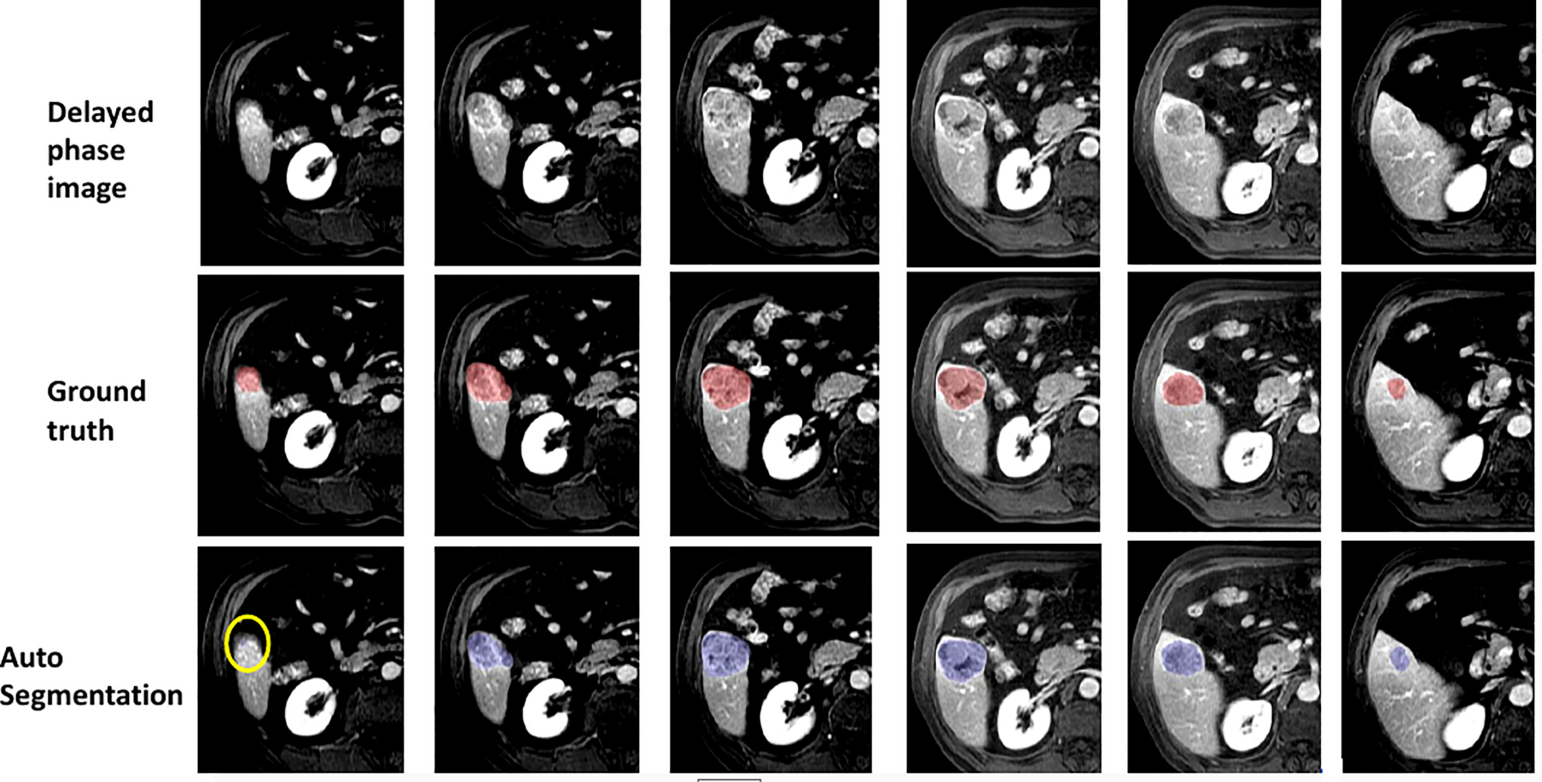
Some examples of segmentation results.

**Figure 8** visually shows the segmentation results of some typical images. The first row is the raw images from the delayed phase. The middle row shows the ground truth with the liver lesion marked in red, and the bottom row displays the results with lesions marked in blue, which are automatically segmented by the algorithm. From **Figure 8**, we can see that the deep learning segmentation model performs well in the last five cases. Although it fails in the first case, the lesion in this case is very challenging. To evaluate the results more quantitatively, various performance evaluation indicators or metrics described above are adopted and utilized in this paper including DICE, precision, recall, sensitivity, and specificity.

**Figure 9:**
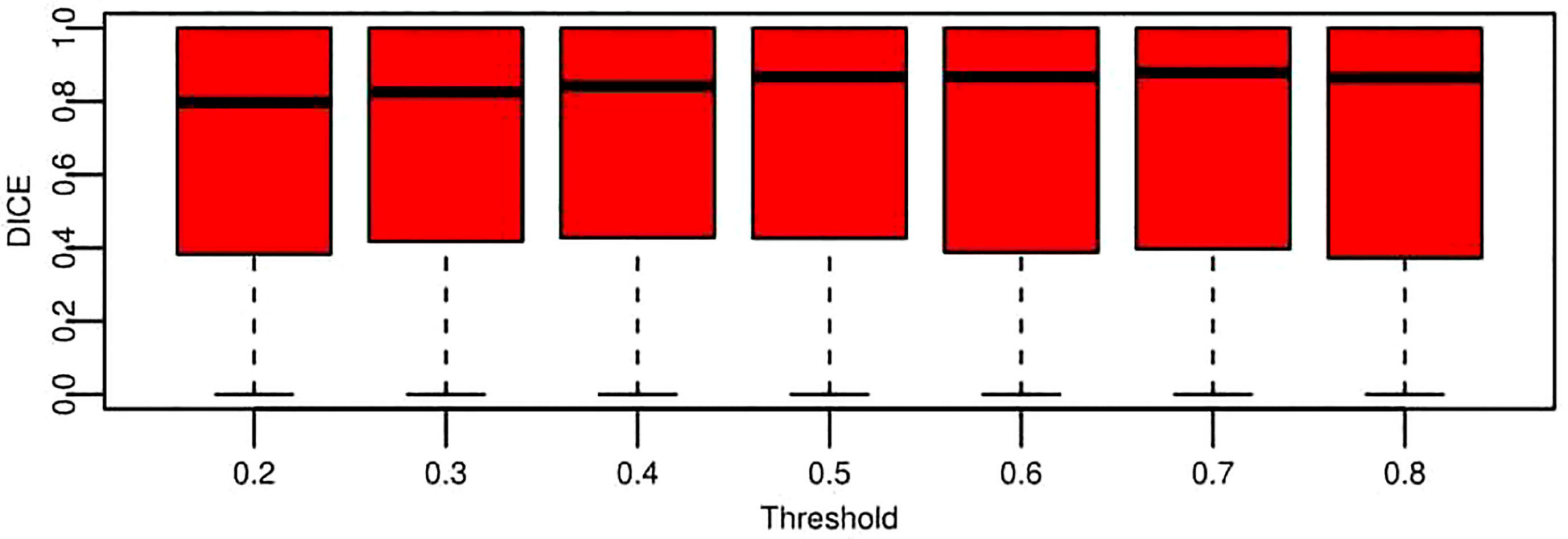
Boxplot for segmentation performance evaluation results based on DICE under the different threshold conditions. These data represent all the DICE values of testing data across nine folds. Each box is for a specific threshold. The thick black lines, boxes, and whiskers denote the median, interquartile range, and 10th and 90th percentiles, respectively.

DICE is a widely accepted evaluation indicator for segmentation-specific tasks, especially in medical imaging studies to quantify the degree of overlap between two binary images that are two segmentation images, namely, model predicted segmentation and segmentation annotated by radiologists. **Figure 9** shows the change of DICE values based on different thresholds being performed on the CNN model predicted segmentation results. To compare the values more intuitively, **Table 4** lists the exact number of average and median DICE values. From these evaluation results, the model accuracy based on testing data from the viewpoint of either median or average is not monotonic, as the threshold increases due to the nature of the DICE definition. This is also in line with the logic that the highest or smallest threshold does not deliver the best segmentation results, and an appropriate threshold (range 0.4–0.7 in this case from **Table 4**) is required to be selected for optimal segmentation results.

**Table 4:** Quantitative comparison of segmentation performance evaluation results based on median and average DICE.

Threshold	0.2	0.3	0.4	0.5	0.6	0.7	0.8
Median	0.798	0.825	0.842	0.867	0.867	0.879	0.864
Average	0.669	0.681	0.688	0.691	0.680	0.687	0.665

**Figure 10:**
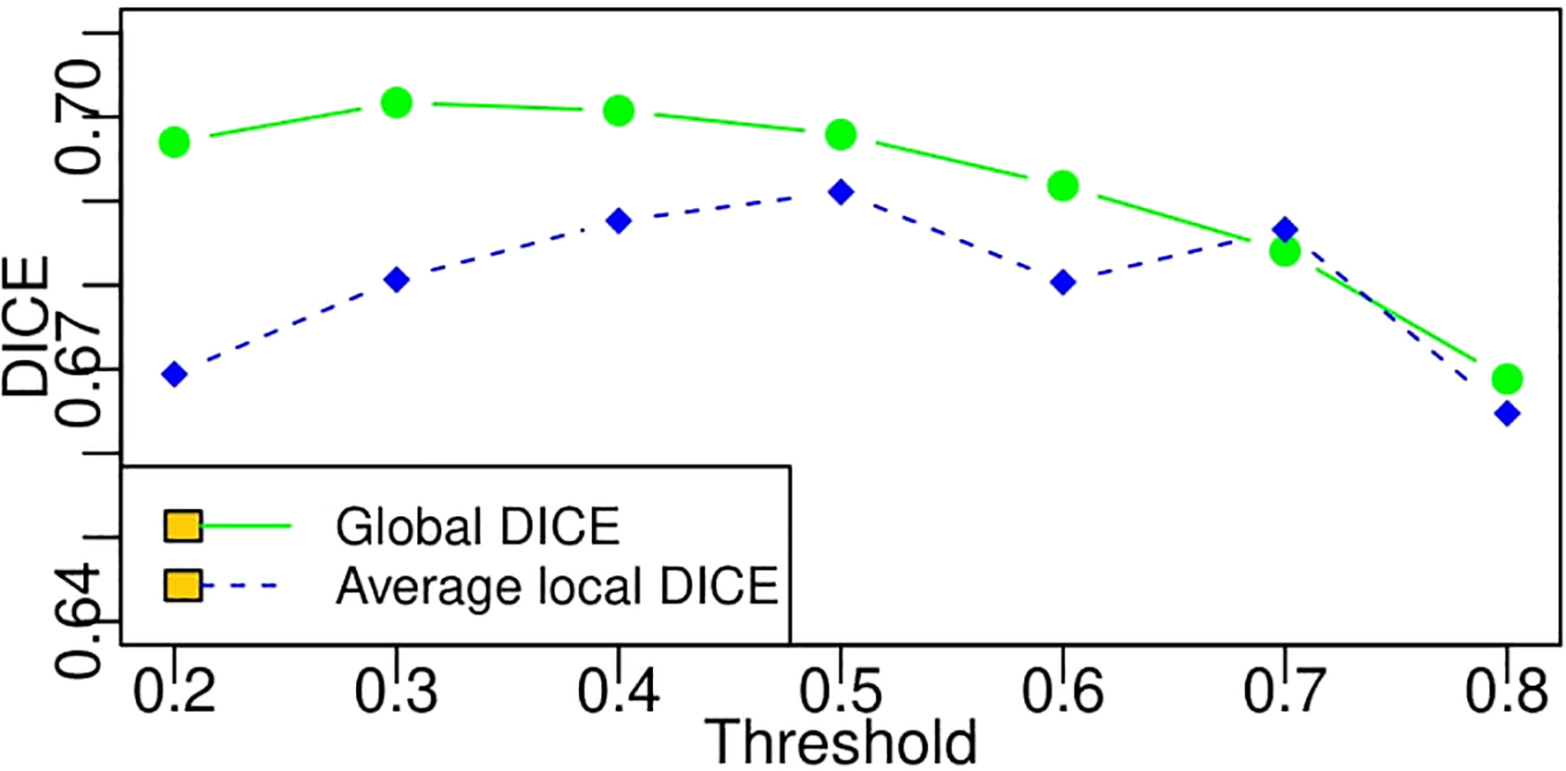
Change curves of average local DICE and global DICE values based on different thresholds. According to the legends in the figure, the green line represents the global DICE, and the blue one indicates the average local DICE, which is the same as the average values in the last line of **Table 4**.

**Figure 11:**
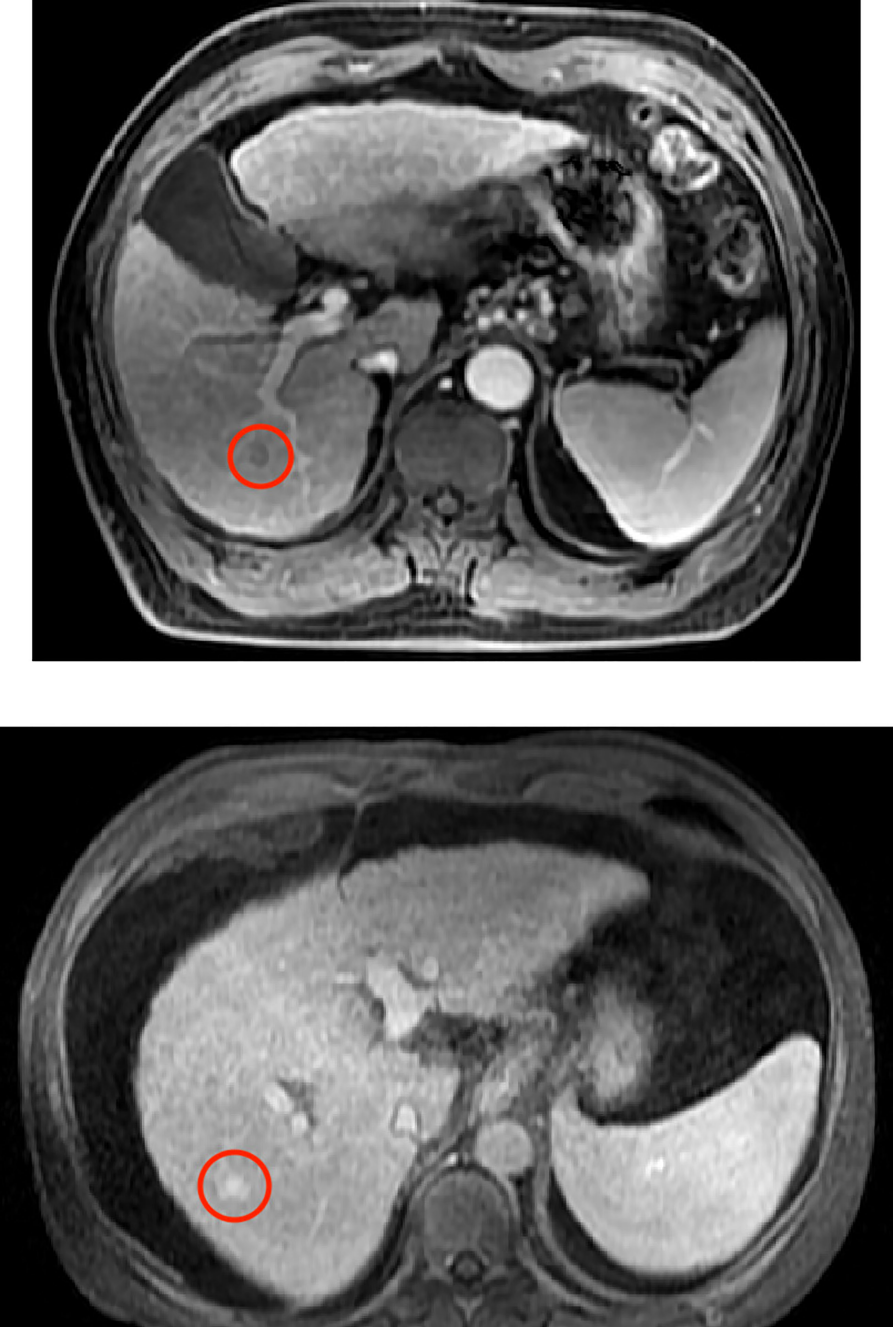
Examples with small lesion sizes. Red circles frame the lesion region identified by radiologists.

The average DICE value based on each threshold in **Table 4** is obtained by averaging the DICE values of all the patients. A DICE value is first calculated for each patient, and the values are averaged into a final value. Moreover, there is another way to compute DICE (global DICE in this paper), which is to average the TN, TP, FN, and FP first and then calculate the final DICE value. Mathematically, average local DICE can be computed by $\frac{1}{n}\sum_{i}^{n}DICE_{i}=\frac{1}{n}\sum_{i}^{n}\frac{2TP_{i}}{2TP_{i}+FP_{i}+FN_{i}}$ , and global DICE is computed by $\frac{2\sum_{i}^{n}TP_{i}}{2\sum_{i}^{i}TP_{i}+\sum_{i}^{n}FP_{i}+\sum_{i}^{n}FN_{i}}$ . **Figure 10** demonstrates the change curves of average DICE values and global DICE values on different thresholds. It can be seen from **Figure 10** that global DICE is generally higher than the average DICE. From observation, this is because the dataset used in this study contains many cases with very small lesion size that is not easy to detect (see examples in **Figure 11**). The lesions with diameter less than 2 cm account for 44.8%. There would be many zeros for the DICE calculation of each patient, which can be observed in **Figure 9** where the whisker that indicates the 10th percentile is near 0. Therefore, when we calculate the average local DICE, the existence of many zeros would drag down the average value.

**Figure 12:**
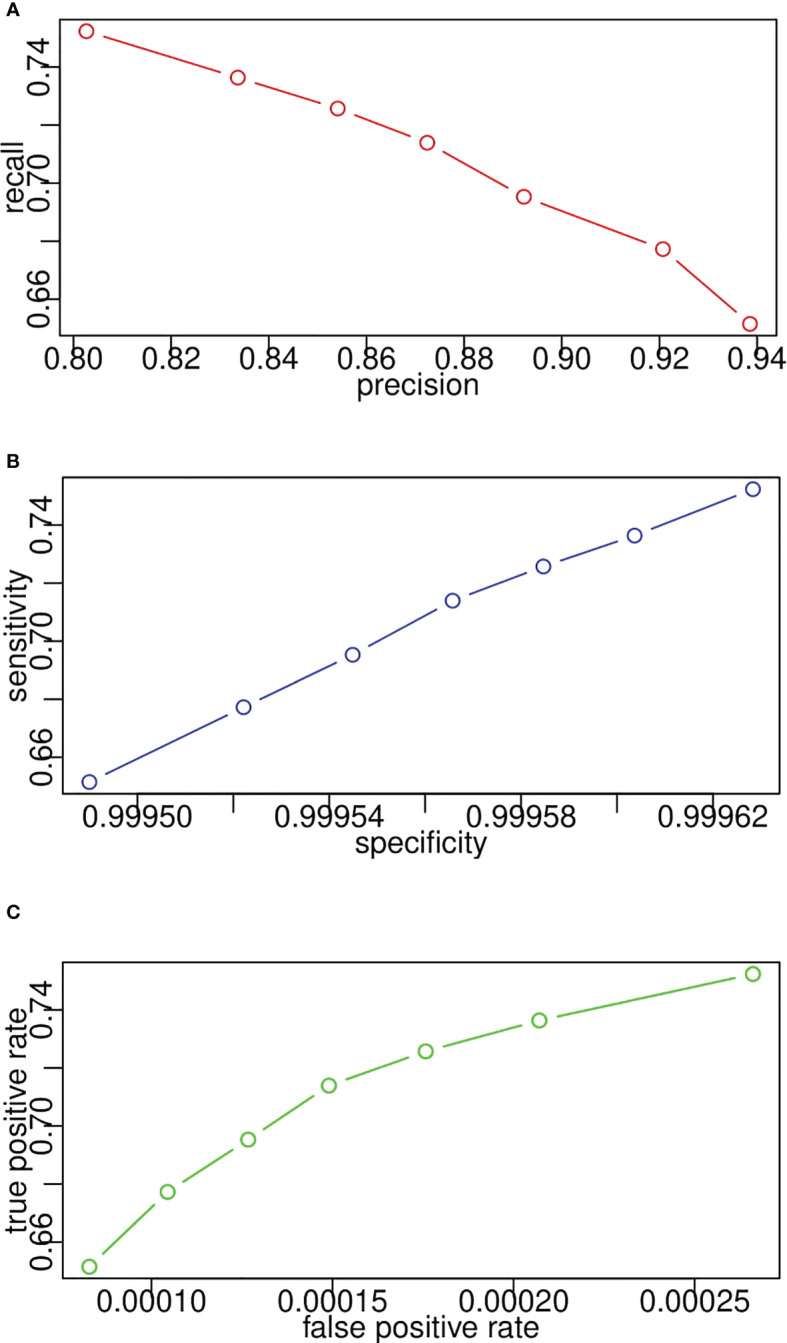
Model receiver operating characteristic curves for lesion segmentation from pixel level in terms of **(A)** recall and precision, **(B)** sensitivity and specificity, **(C)** true positive rate and false positive rate.

**Figures 12A–C** plot the receiver operating characteristic curves from the pixel level in terms of sensitivity and specificity, false-positive rate and true-positive rate, and precision and recall, respectively. Since recall, sensitivity, and true-positive rate represent the same evaluation results according to their calculation equations, the y-axes of **Figures 12A–C** demonstrate the same value. In **Figure 12B**, specificity is consistently high because TN occupies the majority of image pixels. Likewise, in **Figure 12C**, the false-positive rate is consistently low because of the imbalance in the number of lesion pixels and non-lesion pixels. Therefore, we can conclude that precision–recall is enough for performance evaluation, and the other two sets of indicators are not reasonable for segmentation evaluation from the pixel level.

**Figure 13:**
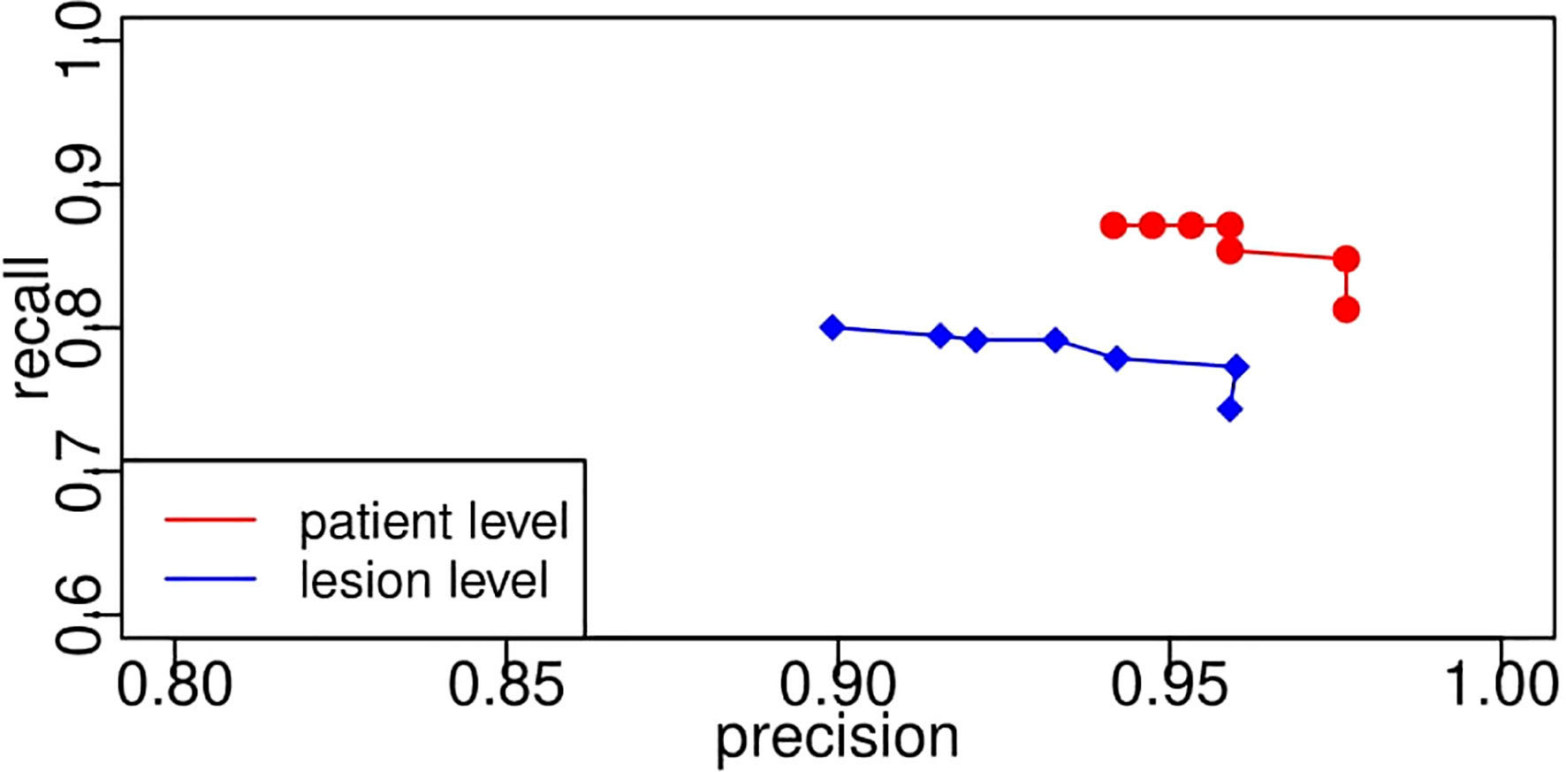
Segmentation model receiver operating characteristic curves based on precision and recall from lesion level and patient level. The PR curve approaching closer to the top-right corner [1.0, 1.0] indicates better performance. PR, precision–recall.

In the case of the pixel level, the number of TP indicates the number of pixels that belong to the lesion and are successfully segmented by the proposed CNN segmentation model. It is easy to compute the values of precision, recall, sensitivity, and specificity from the pixel level given the exact number of TN, TP, FN, and FP. However, it is vague to count the number of them on the lesion level since it is not easy to determine to what extent the segmented lesion could be deemed as a TN. In addition, in the case of patient level, one patient may have multiple lesions, and we need to consider when to determine a patient as a TN. In this study, once a lesion is segmented, it would be treated as a TN. Regarding the patient level, the patient with any lesion being detected would also be a TN. Likewise, **Figure 13** displays the receiver operating characteristic curves based on precision and recall from lesion level and patient level. A threshold is used to transform the intermediate probability maps into binary segmentation results. When thresholds change, the obtained binary segmentation results might also change, thus leading to a change in performance evaluation such as precision and recall. From the plots, as the threshold varies, recall decreases with the increase in precision. As seen, the recall/sensitivity changes within a range of 0.7 to 0.9, while the precision always stays high with a value greater than 0.9. Take the plots as a whole, the evaluation results from the patient level marked in red are better than the results from the lesion level in blue, much closer to [1.0, 1.0]. This exactly meets the expectation of radiologists. From the requirements, it would be enough and great if the framework can help identify the patient who potentially has the lesion. In light of the above quantitative and qualitative analyses, the developed deep learning segmentation model performs well even in our challenging PUK1 dataset.

**Figure 14:**
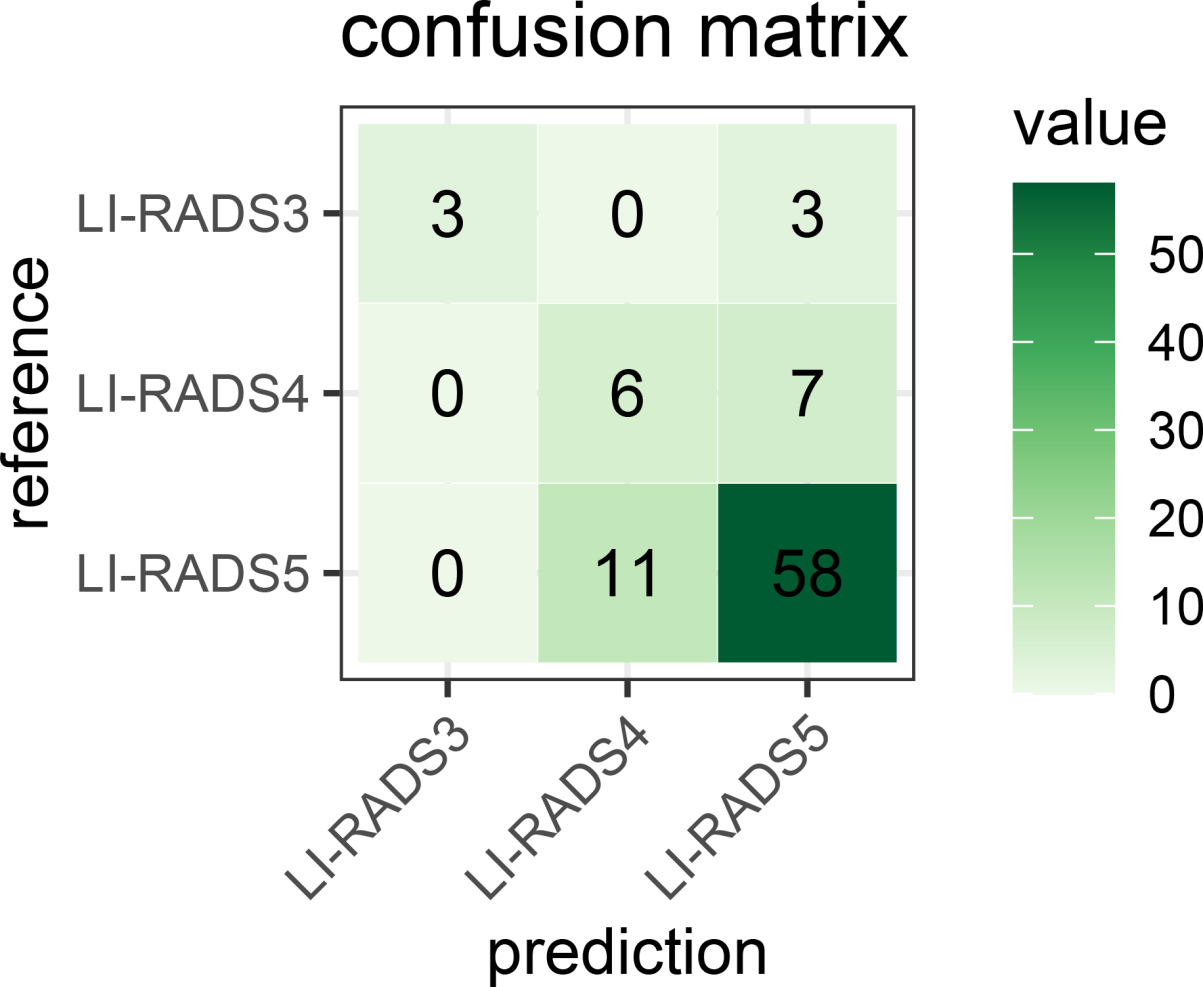
Confusion matrix for the developed LI-RADS category model on the PKU1 dataset. It fully demonstrates the number of predicted each LI-RADS category and true labels. The numbers of correct category predictions are listed on the diagonal line from the top-left corner to bottom-right corner. LI-RADS, Liver Imaging Reporting and Data System.

For LI-RADS scoring, the combination model of lesion feature characterization and inference module is evaluated to have an overall accuracy of 76%. **Figure 14** shows the confusion matrix for LI-RADS 3/4/5 scoring of each category. Overcategorization and undercategorization predictions are displayed in the top-right and bottom-left triangles, respectively. As seen, the most mis-classification cases happen in the neighbor categories mainly because it is very difficult to distinguish LI-RADS 4 from LI-RADS 5.

To demonstrate the significance of the proposed approach, we have searched the recent LI-RADS research papers that use deep learning techniques to achieve the recognition of LI-RADS grade based on MRI. When searching, we applied a selection strategy as 1) the purpose of the paper is to achieve the classification in relation to the LI-RADS score, 2) the method used is deep learning-based, and 3) the input for diagnosis is limited to MRI. As a result, we obtained the six most recent and related papers as listed below, which include the published year and journal of each paper, the information about inputs and outputs, techniques used, and whether they have compared with the state-of-the-art (SOTA).

**(1) References:** Hamm et al. (5)**;**

- **Published journal:** *European Radiology*;
- **LI-RADS classes (outputs):** HCCs (corresponding to LR-5), benign lesions (grouping cysts, hemangiomas, and FNHs, corresponding to LR-1), and malignant non-HCC lesions (grouping ICCs and CRC metastases, corresponding to LR-M);
- **inputs:** 3D bounding box around target lesion (manually cropped);
- **Research purpose:** to develop and validate a proof-of-concept CNN-based deep learning system (DLS) that classifies common hepatic lesions on multi-phasic MRI;
- **Technique used:** a general CNN model (three convolutional layers + two maximum pooling layers + two fully connected layers);
- **SOTA:** no.

**(2) References:** Wu et al. (13)**;**

- **Published journal:** Annals of Transitional Medicine;
- **LI-RADS classes (outputs):** combined LR-4/LR-5 tumor OR LR-3 tumor;
- **Inputs:** a rectangular tumor box centered on the tumor area (manually);
- **Research purpose:** to develop a deep learning (DL) method;
- **Technique used:** AlexNet + fine-tune;
- **SOTA:** no.

**(3) References:** Oestmann et al. (14)**;**

- **Published journal:** *European Radiology*;
- **LI-RADS classes (outputs):** HCCs (corresponding to LR-5), non-HCCs (corresponding to LR<5);
- **Inputs:** manually recorded to define a 3D bounding box around the lesion;
- **Research purpose:** to provide proof-of-concept for CNN-based classification;
- **Technique used:** customized CNN model;
- **SOTA:** no.

**(4) References:** Yamashita et al. (15)**;**

- **Published journal:** *Abdominal Radiology*;
- **LI-RADS classes (outputs):** LR-1/2, LR-3, LR-4, LR-5;
- **Inputs:** manually crop with square regions of interest;
- **Research purpose:** to provide a deep CNN model and show the feasibility of CNN for assigning LI-RADS categories;
- **Technique used:** 1) a VGG16 pre-trained network and 2) a custom-made network;
- **SOTA:** no.

**(5) References:** Stollmayer et al. (16)**;**

- **Published journal:** World Journal of Gastroenterology;
- **LI-RADS classes (outputs):** focal nodular hyperplasia (FNH), hepatocellular carcinoma (HCC), and liver metastases (MET);
- **Inputs:** manually crop;
- **Research purpose:** to compare the performance of 2D and 3D-DenseNets in the classification of three types of FLLs, including FNH, HCC, and MET;
- **Technique used:** DenseNet;
- **SOTA:** no.

**(6) References:** Trivizakis et al. (17)**;**

- **Published journal:** IEEE Journal of Biomedical and Health Informatics;
- **LI-RADS classes (outputs):** discriminate between primary and metastatic liver cancer;
- **Inputs:** not applicable;
- **Research purpose:** to compare 3D CNNs with 2D CNNs;
- **Technique used:** four-strided 3D convolutional layers and one fully connected layer with 2,048 neurons and 50% dropout;
- **SOTA:** no.

**Figure 15:**
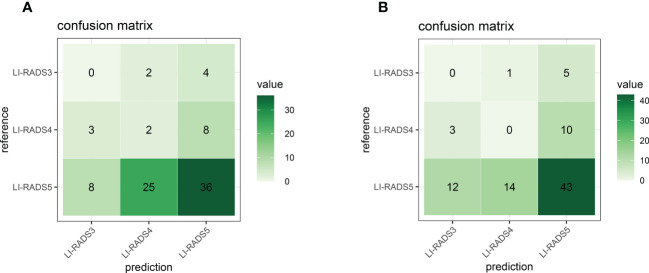
Confusion matrix for the VGG classification model **(A)** without transfer learning and **(B)** with transfer learning.

From the above overview, we can see that current works still stay in the exploration stage and attempt to prove the feasibility of deep learning-based classification in clinical practice by directly adopting popular CNN models in the field of computer vision. None of them has ever tried to compare with SOTA, or it is just because there are not yet well-accepted SOTA algorithms for this specific task. Since the key innovation of our proposed deep learning architecture focuses on the added feature characterization step in comparison to previous related research, we additionally conduct an experiment using basic VGG for LI-RADS classification as the above references did to prove the effectiveness of the added step. The results from VGG with both pre-trained fixed parameters and fine-tuned adjusted parameters are presented in **Figures 15A, B**, respectively. On the PKU1 dataset, pre-trained VGG with and without transfer learning has an overall accuracy of 49% and 43%, respectively. On the one hand, this comparative experiment confirms the expectation that the CNN model with transfer learning outperforms the one without transfer learning for the LI-RADS classification application. On the other hand, it also demonstrates that our proposed approach with the added feature characterization step could significantly improve the accuracy performance in comparison to a CNN architecture as a classification tool. The other reason for such poor performance is that the PKU1 dataset itself is challenging. This also shows that there are still big demands on designing and developing more specific deep learning techniques for LI-RADS rather than simply adopting a classification model that is initially developed for other applications before computer-aided LI-RADS diagnosis can be really used in clinical practice.

**Figure 16:**
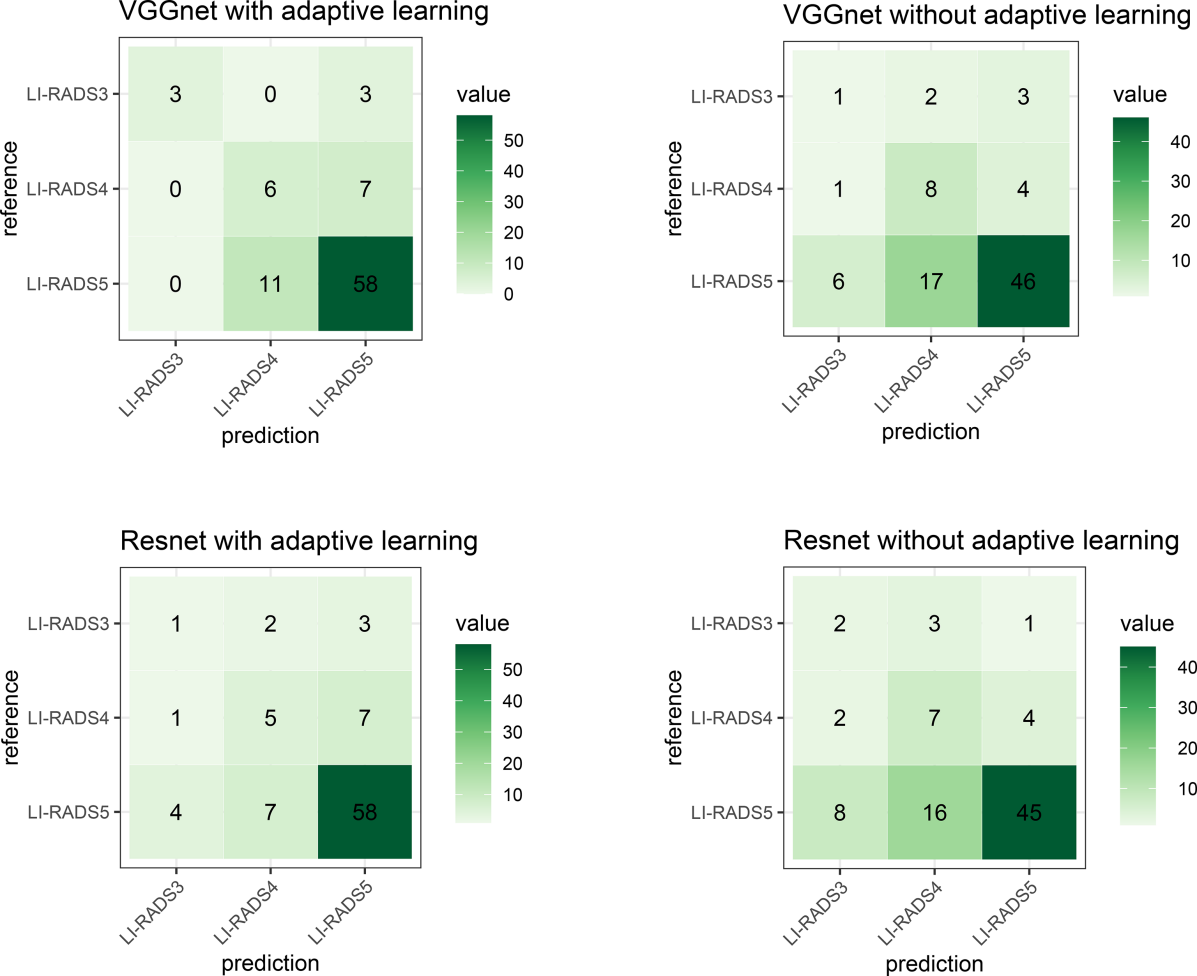
The confusion matrix describes the classification model performance for each combination. The top-left shows the technique used in our study and is similar to **Figure 14**.

As described in Lipton and Steinhardt (18), since sometimes a number of proposed techniques together achieve a significant empirical result, an ablation study was advocated to identify the source of empirical gains; i.e., what really worked? In this study, we are not combining a number of fancy techniques into a complex model as our approach. The key innovation of this manuscript is adaptive learning for the added feature characterization step. Thus, the ablation study in this paper is designed to validate whether adaptive learning works or not. In order to achieve the purpose of an ablation study to elucidate which techniques really contribute to performance improvement, experiments using different technique combinations are separately conducted. The combinations are VGG with and without adaptive learning and ResNet with and without adaptive learning. The confusion matrices of these models are demonstrated in **Figure 16**. To intuitively compare the experiment results, **Table 5** shows the accuracy of their classification performance. Among the four combinations, whether VGGNet or ResNet, they both have better performance when using adaptive learning than without adaptive learning. Comparing VGGNet and ResNet, the difference in the CNN model does not result in big differences in classification performance, although the accuracy on VGGNet is always slightly higher than on ResNet. The main reason should be the overfitting learning of ResNet in this specific case study.

**Table 5:** The accuracy performance of each model using different combinations.

Technique combination	Accuracy
VGGNet with adaptive learning (Ours)	76.13%
ResNet with adaptive learning	72.72%
VGGNet without adaptive learning	63.50%
ResNet without adaptive learning	61.36%

## 4 Discussion

This study provides a fully automatic solution for computer-aided LI-RADS scoring, integrating deep learning-based liver lesion segmentation, lesion feature characterization, and LI-RADS score prediction into one ecosystem. From the list of relevant references, most current existing deep learning-based LI-RADS classification works manually crop the sub-images covering lesion regions that are used to be fed into the classification model. Therefore, they are not fully automatic computer-assisted processes. Compared with the general classification task, the identification flow of lesion types in LI-RADS has unique characteristics. We notice that radiologists can explicitly utilize the reasoning ability to infer the values of several key features before outputting a final LI-RADS score by observing the images from different phases. There are normally three phases that matter greatly for radiologists to diagnose, namely, arterial phase, portal venous phase, and delayed phase. They are captured for the duration of several different time periods after the contrast injection. As mentioned previously, the key features officially identified and defined in LI-RADS consist of APHE, the size of the lesion, the presence of enhancing capsule and non-peripheral washout, and threshold growth. While the domain knowledge of such features plays a vital role in LI-RADS scoring, previous methods focus on directly classifying the types based on a single phase or image. How to endow the existing methods with the capability of multi-phase reasoning and incorporate the LI-RADS domain knowledge into the workflow of deep learning-based classification is vital for automating the LI-RADS system but still remains a boundary to explore. Motivated by the above discussions, this paper aims to propose a complete and automatic solution for LI-RADS scoring that is embedded with a multi-phase reasoning task for discriminative lesion feature characterization, and the newly designed CNN architecture can be jointly trained by using multi-task learning with adaptive loss.

It is a common procedure for image classification tasks to first do the segmentation and then classification. The innovation and key contribution of this paper is not just proposing such a deep learning-based liver segmentation framework but embedding the expert domain knowledge into the deep learning framework and the attempt on practical applications of this framework. Based on the overview of prior relevant research as listed previously, they usually adopted the CNN technique to take lesion region as inputs and output a LI-RADS level (Hamm et al. (5); Wu et al. (13); Oestmann et al. (14); Yamashita et al. (15)) or lesion type (Stollmayer et al. (16); Trivizakis et al. (17)). This paper proposes to introduce one more step, which is called feature characterization, before LI-RADS score classification (see **Figure 1**) and focuses on delving into the multi-phase reasoning problem for discriminative lesion feature characterization. This is our key contribution. By doing so, it is able to unbox the black box for the proposed algorithm and improve the explainability. To solve such multi-phase reasoning problem, this paper develops a new network architecture that utilizes the convolutional layers of the VGG 16 network as the backbone and is integrated with the switch mechanism and multi-task learning strategy. Specifically, multi-task means the characterization of multiple features, namely, the presence of APHE, washout, and capsule.

To validate the performance of the proposed framework, the experiment evaluation is in two parts, namely, segmentation of liver lesion and classification of LI-RADS score. For the segmentation part, we have showcased the visual comparison of the segmentation result with ground truth and evaluated the performance quantitatively based on DICE, precision, recall, sensitivity, and specificity. Thus, the logic of section results is 1) visual demonstration on segmentation; 2) performance on DICE; 3) ROC analysis at the pixel level, lesion level, and patient level; 4) confusion matrix for LI-RADS performance. We presented the average DICE performance index value in two ways (we call global DICE and local DICE in this paper), analyzed their difference, and traced the root cause of such difference, which is the poor performance of lesions less than 2 cm. However, in our study, the test dataset contains approximately 45% of such cases. This analysis result reveals one of the limitations of the proposed algorithm. In addition to the DICE index, ROCs from the pixel level, lesion level, and patient level based on sensitivity and specificity, false-positive rate and true-positive rate, precision, and recall were also considered, and their applicability was discussed. It has been explained if something is not applicable or reasonable. Finally, a diffusion matrix is presented to demonstrate the performance of LI-RADS scoring. In addition, a comparative experiment with and without the feature characterization step has been conducted to show that this added step with multi-task learning really facilitates the diagnostic process.

Although this study provides a proof-of-concept for deep learning-assisted LI-RADS categories and develops an approach from a feature characterization perspective, it still has several limitations. First, the generalization capability of the proposed algorithm needs to be validated further in multiple center populations. Further improvement may be achieved by additional data collection including more institutions and increased diversity in data distribution regarding age, sex, lesion types, and bias control. In addition, the detection and segmentation of liver lesions involved in the proposed automatic framework directly utilize U-net++ and could be improved by a modified version tailored specifically for the task of liver lesion detection. These would be the direction of our future research work by conducting more clinical trials for algorithm software validation and exploring if there is any improvement in lesion detection.

To test the proposed system, patient data with liver lesions are collected and then used to build a challenging PKU1 dataset. More samples will be collected in the future and used to update the system to make it more powerful. In this study, deep learning methods with transfer learning are adopted for both segmentation and classification. Particularly for lesion feature characterization, the pre-trained network is retrained based on the PKU1 dataset using the strategy with adaptive loss function to learn the imaging features. In comparison to previous works as listed, which simply adopted the popular CNN models, although this paper tries to take a step forward to modify and propose a new framework specifically tailored for the LI-RADS scoring task, it is still the first step for the exploration in deep learning-assisted LI-RADS categories. We anticipate that this paper will be used to show that deep learning for LI-RADS scoring is of great potential and practical significance. More research attention should be given to the field of this study, and more techniques could be developed in the coming years.

## Data availability statement

The data analyzed in this study is subject to the following licenses/restrictions: The datasets presented in this article are not readily available because the datasets are privately owned by Peking University First Hospital and are not made public. Requests to access these datasets should be directed to wangxiaoying@bjmu.edu.cn.

## Ethics statement

The studies involving human participants were reviewed and approved by Ethics Board of Peking University First Hospital. Written informed consent for participation was not required for this study in accordance with the national legislation and the institutional requirements.

## Author contributions

KW wrote the article and collect data. YL wrote and revised the article and designed the methods. HC, WY and ZJ designed the methodology, analysis, and interpretation. XW supervised the whole process. All authors contributed to the article and approved the submitted version.
